# EasyGeSe – a resource for benchmarking genomic prediction methods

**DOI:** 10.1186/s12864-025-12129-0

**Published:** 2025-10-24

**Authors:** Carles Quesada-Traver, Daniel Ariza-Suarez, Bruno Studer, Steven Yates

**Affiliations:** Molecular Plant Breeding, Institute of Agricultural Sciences, ETH Zurich, Universitaetstrasse 2, Zurich, 8092 Switzerland

**Keywords:** Genomic prediction, Genomic selection, Benchmarking, Machine learning, Quantitative genetics, Database

## Abstract

**Background:**

Genomic prediction is a widely used method to predict phenotypes from genotypic data. Advances in both biological and computer science have enabled the generation of vast amounts of data and the development of new algorithms, specifically in the field of machine learning. However, systematic benchmarking of new genomic prediction methods, which is essential for objective evaluation and comparison, remains limited.

**Results:**

Here, we present EasyGeSe, a tool that provides access to a curated collection of datasets for testing genomic prediction methods. This resource encompasses data from multiple species, including barley, common bean, lentil, loblolly pine, eastern oyster, maize, pig, rice, soybean and wheat, representing a broad biological diversity. We filtered and arranged these data in convenient formats, provided functions in R and Python for easy loading and benchmarked several modelling strategies for genomic prediction. Predictive performance, measured by Pearson’s correlation coefficient (*r*), varied significantly by species and trait (*p* < 0.001), ranging from − 0.08 to 0.96, with a mean of 0.62. Comparisons among parametric, semi-parametric and non-parametric models revealed modest but statistically significant (*p* < 1e^−10^) gains in accuracy for the non-parametric methods random forest (+ 0.014), LightGBM (+ 0.021) and XGBoost (+ 0.025). These methods also offered major computational advantages, with model fitting times typically an order of magnitude faster and RAM usage approximately 30% lower than Bayesian alternatives. However, these measurements do not account for the computational costs of hyperparameter tuning.

**Conclusions:**

By standardizing input data and evaluation procedures, this resource simplifies benchmarking and enables fair, reproducible comparisons of genomic prediction methods. It also broadens access to genomic prediction data, encouraging data scientists and interdisciplinary researchers to test novel modelling strategies.

**Supplementary Information:**

The online version contains supplementary material available at 10.1186/s12864-025-12129-0.

## Background

Genomic prediction is widely applied in plant and animal breeding to improve the rate of genetic gain for traits of interest [1]. It uses genomic data to predict the breeding value of individual plants or animals [2]. This information can then be used to make informed decisions about which individuals to select for the next generation/breeding cycle, referred to as genomic selection. Integrating genomic selection ultimately leads to the development of more productive and higher-quality crops and livestock. It not only increases genetic gain for desired traits but also enables optimal allocation of resources, making it a cost-effective strategy for advancing crop and livestock breeding programs [3].

Methods to build genomic prediction models can be divided into three main categories: parametric, semi-parametric and non-parametric [4]. Parametric methods include genomic best linear unbiased prediction (GBLUP) and Bayesian methods such as BayesA, BayesB, BayesC, Bayesian lasso (BL) and Bayesian Ridge Regression (BRR) [4]. The mostly used semi-parametric method for genomic prediction is the reproducing kernel Hilbert spaces (RKHS) [5], which uses the Gaussian kernel function to fit the model. Non-parametric methods are usually machine learning algorithms. Widely used machine learning algorithms for genomic prediction include random forest (RF) [6], support vector regression (SVR) [7], kernel ridge regression (KRR) [8] and gradient boosting [9], among others.

Machine learning is a growing discipline that has been gaining popularity across a wide range of research fields. This trend can be attributed to the development of new algorithms and the generation of large quantities of data, both made possible by advances in technology [10]. Genomic prediction is an evolving field that is also benefiting from these advancements, and more machine learning-based modelling strategies can be expected in the future [11]. When these novel methods are proposed to predict phenotypic values from genotypic data, they are often benchmarked only using species-specific study data. However, selecting a single dataset limits the generalizability of the results. This is because of the diversity of biological organisms and traits, each with their own unique characteristics that cannot be captured in a single dataset from one species. Different species have different reproduction systems, genome sizes, ploidy levels and chromosome numbers, all of which influence the breeding scheme and the accuracy of genomic prediction models. Thus, the transferability of insights and the adoption of novel approaches across species and populations remains limited. Yet, in other fields, benchmarking datasets are commonly available for testing new modelling approaches: for instance, Imagenet [12], which was instrumental in driving computer vision. Despite an increasing amount of data [11] and requirements for publicly funded experiments to be findable and accessible [13], to the best of our knowledge, such a resource is not available for genomic prediction.

There is a need to have a tool that gathers publicly available datasets that are clean and in a ready-to-use format to be tested by experts from all fields (biologists, bioinformaticians and data scientists). We propose assembling a representative range of data to test novel modelling strategies for genomic prediction. The benefits of such a tool allow consistent and comparable estimates of accuracy, from which inferences may be drawn to other datasets. Given the adoption of genomic prediction by scientists and breeders alike, it is vital that data is available for education and testing. However, even publicly available datasets often represent practical barriers to use: links may be broken, files incomplete and formats not consistent, ranging from tab- or comma-separated files, or sequence-based data as a variant call format (VCF) [14], to hierarchical data format version 5 (HDF5) [15], and novel structures. For example, the genotypic data in this resource originated from four different formats. To overcome this, EasyGeSe presents data from multiple sources arranged in convenient formats that are easy to load for analysis and exploration.

## Construction and content

### Data sources

**Fig. 1:**
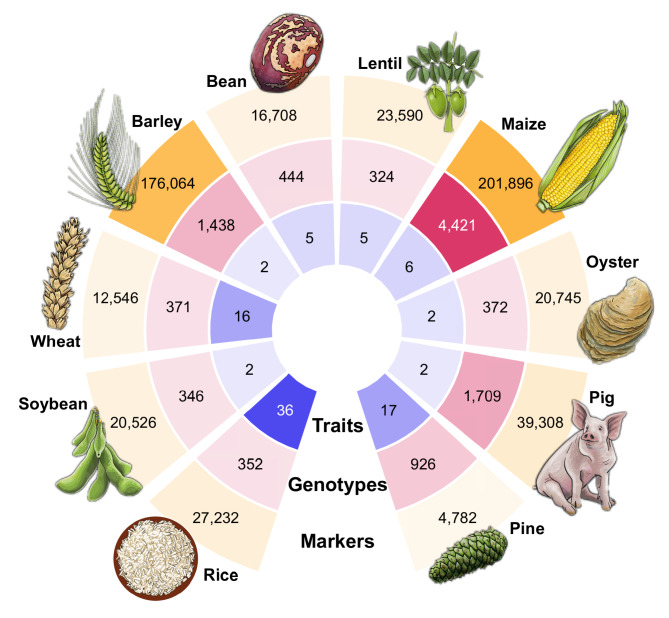
Summary metrics for the data included in EasyGeSe. From the inside to the outside, the polar plot shows the number of traits (blue), number of individuals (red) and number of markers (gold) for the ten species used. Color opacity within each segment reflects the relative magnitude of the corresponding values

The data contained in EasyGeSe was drawn from ten studies and is summarised in Fig. 1, Table 1 and described below. These studies were chosen to account for a wide range of biological diversity, agronomic importance and genetic representativeness and, critically, the data was publicly available. Except stated otherwise, phenotypic data was used as provided by the original authors.

**Table 1:** Characteristics of the EasyGeSe datasets. The table includes the final number of individuals, traits and markers. The table also includes the number of (raw) markers obtained from the original files, together with the final number of markers used after filtering and the number of imputed markers and overall percent of missing data. Also included are the species attributes: number of chromosomes, ploidy level, breeding type, genome size and population structure

Species	Individuals (*n*)	Raw marker (*n*)	Imputation method	Final marker (*n*)	Imputed markers (*n*)	Missing markers (%)	Traits (*n*)	Chromosomes (*n*)	Ploidy	Mating/Breeding system	Population structure	Genome Size (Mb)
Barley	1,438	306,050	Beagle	176,064	8,628,724	3.49	2	7	2	Inbreeding	Genebank accessions	4,540
Bean	444	20,615	Beagle	16,708	1,559,168	21.02	5	11	2	Inbreeding	MAGIC	587
Lentil ^*^	324	30,569	Beagle	23,590	16,298	0.21	5	7	2	Inbreeding	Diversity	4,117
Loblolly pine	926	4,853	SVD	4,782	170,472	3.79	17	12	2	Outbreeding	32 parent, 61 family	21,600
Maize^**^	4,421	437,214	Beagle	201,896	14,940,304	1.67	6	10	2	Hybrid breeding	Hybrid, from multiparent	2,365
Oyster	372	27,407	Beagle	20,745	279,842	2.74	2	10	2	Outbreeding	Four F_2_ families	685
Pig	1,709	40,016	Beagle	39,308	237,517	0.35	2	19	2	Outbreeding	Landrace and hybrid	2,530
Rice	352	36,850	Beagle	27,232	466,931	4.87	36	12	2	Inbreeding	Diversity	430
Soybean	346	31,260	Beagle	20,526	2,009	0.03	2	20	2	Inbreeding	Diversity	1,105
Wheat	371	18,147	SVD	12,546	62,859	0.93	16	7	6	Inbreeding	Diversity	14,540

^*^ The authors of the lentil data reported imputed data using the population mean per marker

^**^For the maize data, the parental data of the hybrids was imputed

#### Barley (Hordeum vulgare L.)

The data is based upon winter barley accessions from the Leibniz Institute of Plant Genetics and Crop Research (IPK) Genebank [16]. The phenotypic data is for disease resistance to barley yellow mosaic virus (BaYMV) and barley mild mosaic virus (BaMMV). Mosaic symptoms were visually scored on a scale from 1 to 9, where 1 means no disease and 9 denotes completely susceptible. The given phenotypic data is the best linear unbiased estimator of historical screenings from 1985 to 2006. It includes data from 1,751 accessions that were genotyped using genotyping-by-sequencing [17]. The genomic data were filtered to remove single nucleotide polymorphisms (SNPs) with a minor allele frequency (MAF) below 5% or with more than 10% missing data. After filtering and imputation, the resulting number of SNPs was 176,064.

#### Common bean (Phaseolus vulgaris L.)

The data comes from a multiparent advanced generation inter-cross (MAGIC) population, developed from eight Mesoamerican breeding lines originating from the Mesoamerican bean breeding program of the International Center for Tropical Agriculture (CIAT) [18]. In total, data is presented from 444 lines for five traits: yield (Yd, kg ha^−1^), days to flowering (DF, days), days to physiological maturity (DPM, days), seed weight (100SdW, g 100 seed^−1^) and pod harvest index (PHI, %) measured in a field trial in 2013. The plants were genotyped using genotyping-by-sequencing [17]. The original matrix used the following filtering: genotype quality above 40, MAF above 0.05, and at least 260 individuals genotyped per site. After Beagle imputation, duplicated marker calls were removed, leaving 16,708 polymorphic SNPs of the original 20,615 SNPs.

#### Lentil (Lens culinaris Medik.)

The data is derived from a lentil diversity panel [19] composed of 324 accessions obtained from the International Center for Agricultural Research in the Dry Areas (ICARDA), the United States Department of Agriculture (USDA), Plant Gene Resources of Canada (PGRC) and the Crop Development Centre (CDC) at the University of Saskatchewan. The data includes five traits: days to flowering (DTF, days), vegetative period (VEG, days), days to swollen pods (DTS, days), days to maturity (DTM, days) and reproductive period (REP, days). The accessions were genotyped using a custom exome capture assay as described by Ogutcen et al. [20] with 23,590 SNPs. No filtering or imputation were made in this study as the data was complete, and the authors already applied a MAF filtering of 0.05.

#### Loblolly pine (Pinus taeda L.)

The data originates from 61 families (from 32 parents) and contains measurements from 926 trees, phenotyped for 17 traits [21]. The traits include stem diameter (DBH, cm), total stem height (HT, cm) and total height to the base of the live crown (HTLC, cm), crown width across the planting beds (CWAC, cm), crown width along the planting beds (CWAL, cm), basal height of the live crown (BLC, cm), branch angle average (BA, degrees), average branch diameter (BD, cm), tree stiffness (stiffnessTree, km^2^/s^2^), lignin content (lignin), latewood percentage at year 4 (lateWood.4), wood specific gravity (density) and 5- and 6-carbon sugar content (c5c6Gall volume, gall) and absence or presence of rust (rustbin), two traits related to fusiform rust susceptibility, are also included. Additionally, from a rooting experiment under greenhouse conditions, root number (rootnum) and presence or absence of roots (rootnumbin) are also reported. The plants were genotyped with an Illumina Infinium assay [22] (Illumina, Inc, San Diego, CA, USA), which yielded 4,853 polymorphic markers. Given the population design with 32 parents, no MAF filtering was applied to preserve markers specific to one or few parental genotypes, as in the original study. Only duplicated marker calls were removed, leaving 4,782 markers.

#### Eastern oyster (Crassostrea virginica)

The data is from four F_2_ families exposed to acute low salinity [23]. The individuals were exposed to 2-day salinity step down with increased temperatures (to mimic when coastal waters are flooded by heavy rainfall, which lowers costal sea water salinity). Length (mm, mm) and day to death (daydead, day) were recorded in a 36-day experimental setup. In total, 372 oysters were genotyped using doubledigest restriction association DNA sequencing [24]. No filtering was made for MAF or missing data in this study, as the authors previously filtered markers with MAF below 5%. Only duplicated marker calls were removed, leaving 20,745 SNPs from the initial amount of 28,638 SNPs.

#### Maize (Zea mays L.)

The data is derived from the training set of the Genomes to Fields (G2F) 2022 Prediction Competition [25]. The data includes best linear unbiased estimators of 4,422 (out of 4,638) maize hybrids for six traits, which are based upon 217 independent field trials across eight years (2014 to 2021). The phenotypic data includes yield (Yield_Mg_ha, Mg/ha), plant and ear height (Plant_Height_cm and Ear_Height_cm, cm), pollen and silking (Pollen_DAP_days and Silk_DAP_days, days) and grain moisture (Grain_Moisture, %); the data are zero-centred. Best linear unbiased estimators were calculated using a mixed-linear model with the function “lmer” in the R package ‘lme4’ (v1.1–35.5.5). Both hybrid and trial (environment × trial) were used as random effects in the mixed model. The best linear unbiased estimators were extracted using the “ranef” function in R. Whilst the original data contained 437,214 SNPs, after filtering for MAF and missing data using vcftools (v0.1.15, --maf 0.05 --max-missing 0.90) and removing duplicates, 201,896 SNPs were retained.

#### Pig (Sus domesticus)

The data is from a genome-wide association study evaluating intramuscular fat content in 1,709 pigs [26]. The pigs were sampled from landrace, Yorkshire, landrace and Yorkshire hybrid and Duroc × landrace × Yorkshire hybrid populations The two traits measured include marbling score (MS, mean score from three meat quality scoring panellists) and proportion of fat areas in the image (PFAI, based on image segmentation). The pigs were genotyped using the CC1 PorcineSNP50 BeadChip (Illumina), of which 39,308 non-redundant SNPs were retained after removing duplicates from the original 40,016 markers. No filtering was made for MAF or missing data in this study, as the original authors previously applied a 5% MAF filtering. 

#### Rice (Oryza sativa L.)

 The data comes from a diversity panel study [27]. The phenotypic data includes 34 traits: flowering time at Arkansas (days), flowering time at Faridpur (days), flowering time at Aberdeen (days), flowering time ratio of Arkansas Aberdeen, flowering time ratio of Faridpur Aberdeen, culm habit (°), leaf pubescence (binary), flag leaf length (cm), flag leaf width (cm), awn presence (binary), panicle number per plant (n), plant height (cm), panicle length (cm), primary panicle branch number (n), seed number per panicle (n), florets per panicle (n), panicle fertility (%), seed length, seed width, seed volume, seed surface area, brown rice seed length, brown rice seed width, brown rice surface area, brown rice volume, seed length width ratio, brown rice length width ratio, seed color (0–1, pigmented – non pigmented), pericarp color (0–1, pigmented – non pigmented), straighthead susceptibility (0–9, normal – no panicle emergence), blast resistance (0–9, no disease - dead), amylose content (%), alkali spreading value (2–7) , protein content (%), year 2007 flowering time at Arkansas (days) and year 2006 flowering time at Arkansas (days). Genotypic data for 352 inbred accessions of *O. sativa* with 27,233 markers come from an Affymetrix SNP array (Thermo Fisher Scientific, Waltham, MA, USA) containing 36,850 SNPs, from which 27,232 were retained after filtering for duplicated marker calls. No further filtering was made for MAF or missing data in this study, as the authors previously applied a 1% MAF filtering.

#### Soybean (Glycine max (L.) Merr.)

The data includes 346 soybean lines. The phenotypic data describes canopy wilting [28] (CW), scored using a visual rating based on a scale from 0 (no wilting) to 100 (plant death). In addition, water use efficiency (C13) was measured by isotope analysis of carbon isotope ratio [29] (δ13C), by harvesting the aboveground portion of five plants. Plants were genotyped using the Illumina Infinium SoySNP50K iSelect SNP Beadchip (Illumina), after filtering for duplicated marker calls, 20,526 SNPs were retained. As the authors previously filtered for SNPs with a MAF below 5%, no further filtering was made for MAF or missing data in this study.

#### Winter wheat (Triticum aestivum L.)

The data is a collection drawn from a series of studies using the GABI-WHEAT panel [30], a selection of elite European wheat cultivars (358 winter and 14 spring) released between 1975 and 2007. The agronomic traits include: heading date (HD, days), plant height (PH, cm), thousand grain weight (TGW, g), ear weight (EW, g), grains per ear (GPE, n), yield (YIE, Qt/ha), specific weight (SW, kg/m^3^), grain hardiness (GH, %), starch content (STC, %) and protein content (PC, %), sedimentation test (SDS, ml), Hagberg falling number (HAG, s), Zeleny sedimentation index (ZEL, ml), resistance to fusarium head blight (FHB), resistance to septoria blotch (STB) and resistance to tan spot (DTR), as described in detail by Gogna et al. [30]. The GABI-WHEAT panel was genotyped with the 90k iSELECT Illumina chip (Illumina). The genomic data were filtered to remove SNPs with a MAF below 5.93% (350/371) or with more than 4.58% (17/371) missing data. After removing duplicated marker calls, 12,546 polymorphic SNPs were retained.

### Genotypic data imputation

**Fig. 2:**
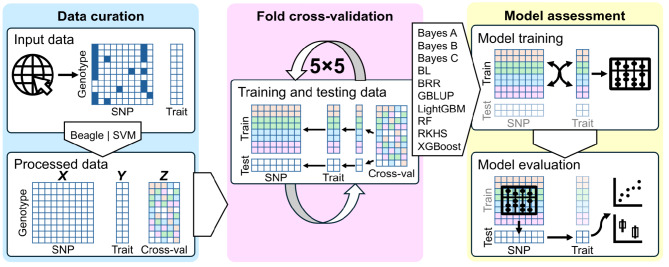
Workflow diagram showing data processing and technical validation. The workflow is organized into three sections: data curation, fold cross-validation and model assessment. Input data originate from publicly available datasets. These data are filtered for missing values and minor allele frequency, after which missing values are imputed using Beagle software or SVM. The processed data are assembled into two matrices: X (genotypic data) and Y (phenotypic data). A novel Z matrix is also created, detailing a 5×5-fold cross-validation scheme in which each trait, per species, is assigned to one of five folds for five independent cross-validations. This scheme allows consistent assignment of data for training and testing purposes. The resulting datasets can be used for benchmarking with prediction algorithms (such as those detailed in this study), where models are trained using the training data and their performance is evaluated against the testing data. Evaluation metrics include correlation and root mean square error, which are in turn used to assess model performance using each fold as an independent measurement, analyzed via linear models

After compiling all the datasets, they underwent a common processing pipeline. An overview of the data processing and technical validation is given in Fig. 2. For datasets containing positional data (chromosome and base-pair position), genotype imputation was performed using the Beagle software (v5.4) [31]. For datasets lacking genome position information (pine and wheat), missing genotypes were imputed using singular value decomposition (SVD) [32, 33]. Initially, missing values were temporarily filled with a zero and the data was decomposed into a matrix of singular values, a matrix of left singular vectors and a matrix of right singular vectors using an SVD function. Singular values explaining 0.95 proportion of total variation (based on cumulative sum of right singular vectors) were retained to reconstruct an approximation of the original matrix. The missing genotypic values were then imputed using values from this reconstructed matrix. Information on imputation is given in Table 1.

### Data format

For ease of use, the data was formatted in the same style as the BGLR wheat data [34], which is distributed with the BGLR R package for testing genomic prediction methods. Essentially, the data was split into two matrices: phenotypic data (matrix Y) and genotypic data (matrix X). For a given species, both matrices have the same number of rows and the same row names. They encode the records for each genotype.

In the Y matrix (phenotypic), each column represents one of the traits for each species, coded with the abbreviations previously described in the section “Data Sources”. Some traits contain missing values as “NA”.

In the X matrix (genotypic), each column represents a genetic marker. The columns are labelled using a standardized format with three components separated by underscores (_): the species name, where the genus is capitalized and the species is lowercase (e.g., Hv for *Hordeum vulgare*); the chromosome number, zero-padded to two digits (e.g., 01); and the position, the base-pair location on the chromosome. For example, the marker “Hv_02_11589” refers to *Hordeum vulgare*, chromosome 2, position 11,589 bp. These matrices do not contain any missing values. Allelic forms are coded using the 012 system. For a given marker, a value of 0 indicates that the genotype is homozygous for the reference allele, 1 indicates that the genotype is heterozygous and 2 codes for a homozygous genotype for the alternative allele.

Additionally, a JSON file is provided for each species, where the cross-validation indices can be retrieved for every species-trait combination.

### Representativity analysis

A principal component analysis (PCA) of the datasets included in EasyGeSe was performed to assess biological diversity and representativeness. The parameters used to characterize each dataset included: the scaled (*z*-score) number of individuals, number of markers, number of traits, mating/breeding system (inbreeding, hybrid breeding or outbreeding), the number of chromosomes, genome size and ploidy (Table 1). Collectively, these parameters reflect key biological attributes of each species, which may influence the accuracy of genomic prediction models. To visualize the relationships between datasets and parameter contributions, a loading plot was overlaid on the resulting PCA score plot, generating a biplot.

### Benchmarking strategy

The benchmarking strategy was used to predict genomic estimated breeding values in the case of parametric methods and genetic values (including non-additive effects) in the case of semi-parametric and non-parametric methods. These were predicted from the Y matrix using genetic data from the X matrix.

#### Parametric and semi-parametric methods

Parametric and semi-parametric methods implemented in ‘BGLR’ (v1.1.3) [34] were tested for genomic prediction. The methods included GBLUP, BayesA, BayesB, BayesC, Bayes Lasso and Bayes Ridge Regression. These parametric models estimate the regression coefficients using different types of prior densities for the marker effects, leading to different strategies to model their additive effects. In addition, the Bayesian RKHS regression was tested by fitting a single Gaussian kernel model in the (average) squared-Euclidean distance between genotypes, with a fixed bandwidth parameter $\:h=0.5$ that was arbitrarily chosen. This semi-parametric regression models additive and non-additive effects of the markers. Inferences were based on 10,000 iterations with a thin of 5, obtained after discarding the first 1,000 burn-in iterations. All other BGLR parameters were set to their default values by the software across all species and datasets.

#### Non-parametric methods

Three different tree-based methods were tested. The first method used was the implementation of random forest (RF) regressor from the ‘scikit-learn’ package (v1.6.1). The second method used was the extreme gradient boosting (XGBoost) regressor from the ‘xgboost’ package (v3.0.0). The third method used was light gradient boosting machine (LightGBM) regressor, implemented in the package ‘lightgbm’ (v4.6.0). Hyperparameter tuning was performed using the Bayesian hyperparameter optimizer ‘optuna’ (v4.2.1) for each combination of model-species-phenotype and the hyperparameter space for each modelling strategy is depicted in Table 2. This is a crucial step when fitting machine learning-based models, as it can increase model accuracy compared to using default parameters [35, 36]. Every set of hyperparameters was optimized using 100 Optuna trials, where each trial was evaluated by 5-fold cross-validation and the mean root mean square error (RMSE) across folds was minimized. The hyperparameter set yielding the lowest mean RMSE was selected to fit the final genomic prediction model. All combinations of optimal hyperparameters per species and trait combination are reported in Tables S1-S3. Non-parametric models were tuned and fitted using Python (v.3.8.17).

**Table 2:** Range of hyperparameters tested for running Optuna. If not present in the table, values were left as default

Model	Hyperparameter	Range/Values
Random Forest	n_estimators	50–1,000
	max_depth	3–10
	min_samples_split	2–10
	min_samples_leaf	1–8
	max_features	['sqrt', 'log2']
	bootstrap	[True, False]
XGBoost	n_estimators	50–1,000
	max_depth	3–15
	learning_rate*	0.01–0.3
	subsample	0.5–1.0
	colsample_bytree	0.5–1.0
	min_child_weight	1–10
	gamma	0–1
LightGBM	n_estimators	50–500
	learning_rate*	0.01–0.2
	num_leaves	10–50
	feature_fraction	0.01–0.2
	lambda_l1*	0.1–5.0

* In logarithmic scale

#### Cross-validation strategy

To estimate the performance of the genomic prediction models, a 5 × 5-fold cross-validation strategy was used, i.e., each dataset was divided into five folds, five times. For each group of five folds, one of the folds was used as the validation set and the other four as the training set, amounting to 25 measurements of each performance metric for each combination of modelling strategy and trait. To ensure a comparable estimate of performance between modelling approaches, we used a paired comparison [37] where the performance of all strategies was estimated using identical partitions and number of folds. Although 5 × 5-fold cross-validation may result in smaller training sets for datasets with limited sample sizes, this approach was applied uniformly to maintain comparability.

### Resource usage

Model training and validation were conducted on a machine running Ubuntu 22.04, equipped with AMD EPYC 9654 CPUs (Advanced Micro Devices, Inc., Santa Clara, CA, USA) and 384 GB of RAM. Each of the 5 × 5-fold cross-validation splits for each crop-trait-model was executed on a single CPU thread. The running time to fit each model on every split was measured using the ‘system.time’ function in R (v4.4.0) and the ‘resource.getrusage(resource.RUSAGE_SELF)’ method in Python (v3.8.17). Maximum memory usage (maximum resident set size) and total user time across all 5 × 5-fold cross-validation splits were recorded using the GNU “time” program for each script call.

To identify the factor that best explained running time, linear models were tested running time against number of samples, number of markers or number of data points (samples × markers), with each fold treated as an independent measurement (e.g. summary(lm(time ~ points))). Model accuracy was measured using *p*-value and adjusted-*r*^*2*^.

Model comparison To identify significant differences between species, traits and models, a linear model was fitted with correlation as the dependent variable and species, traits and model as independent variables. Summary data including *p* and *r*^*2*^ were extracted using the ‘summary’ function. All *r*^*2*^ values reported are adjusted *r*^*2*^.

Unless otherwise stated, all data were analysed or processed using R statistical environment (v4.1.3) and plots rendered using “ggplot2” (v3.4.1).

## Utility and discussion

### Assessment of data representativity

**Fig. 3:**
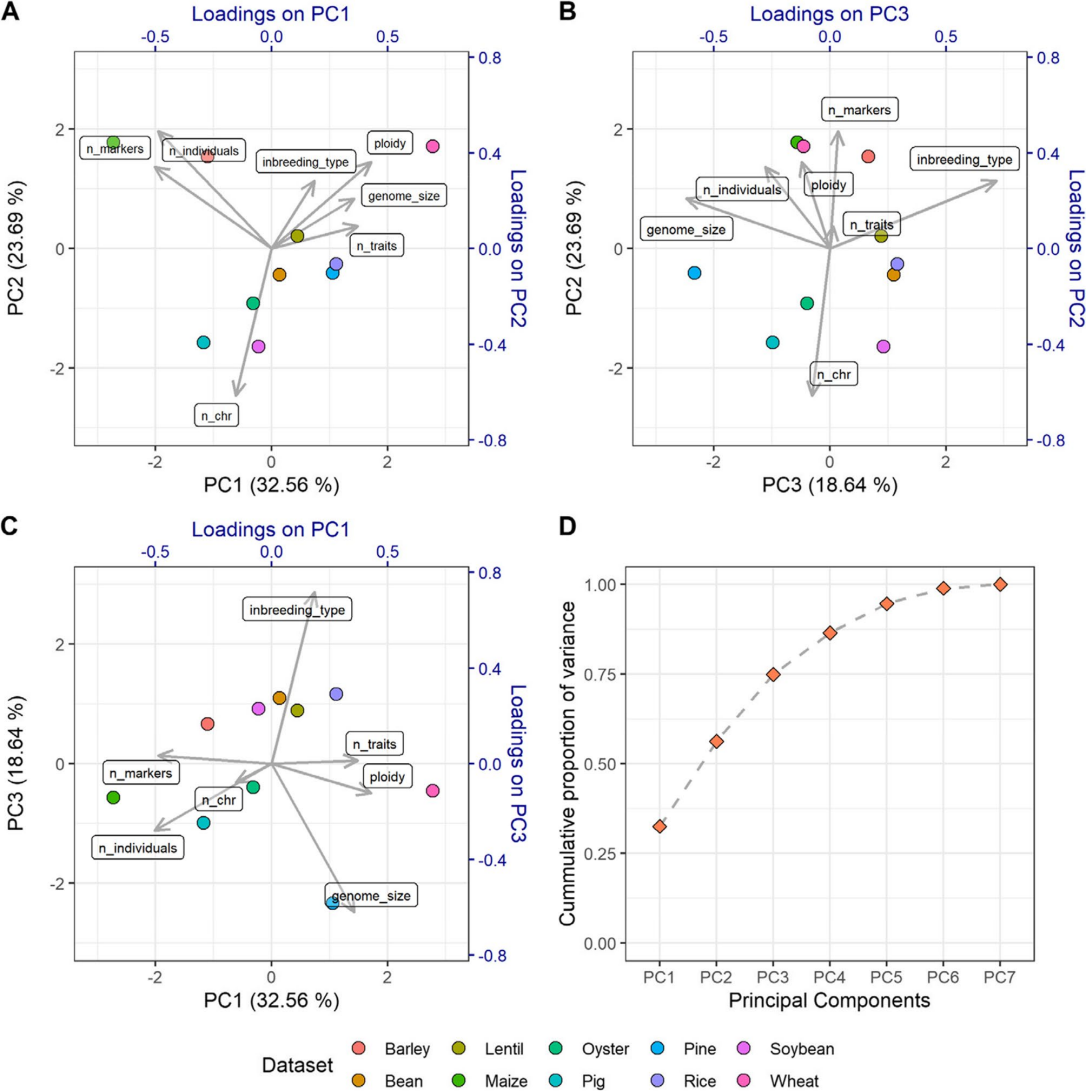
Biplots showing results of the principal component analysis (PCA) on ten datasets (points), based on seven variables. These variables include the number of individuals (n_individuals), number of markers (n_markers), number of traits (n_traits), mating/breeding system (inbreeding_type), number of chromosomes (n_chr), genome size (genome_size) and ploidy level (ploidy). (A–C) Biplots of the first three principal components: PC1 vs. PC2 (A), PC3 vs. PC2 (B), and PC1 vs. PC3 (C). Datasets are represented as coloured points, and variable loadings scaled by a magnitude of four are shown as grey arrows. (D) Cumulative proportion of variance explained by the principal components

Assessment of the data diversity, based on features using PCA, revealed a good separation of the datasets (Fig. 3). Examination of the first three loadings showed little clustering of the datasets and an even spread of the input features (number of individuals, number of markers, number of traits, mating/breeding system, number of chromosomes, genome size and ploidy level), as shown in Table 1. Taken together, these parameters represent to some extent the biology of a species, which may affect the accuracy of genomic prediction.

### Assessment of model correlations

Pearson’s correlation coefficient (*r*), RMSE and user time were measured for every trait and are reported in Table S4. Across traits, *r* and RMSE were strongly negatively correlated (*r = -*0.85) with 71 out of the 93 traits showing *r* < −0.9. Given this strong concordance and the fact that *r* is the preferred metric in genomic selection studies, we focused subsequent reporting on *r*. However, all RMSE values are provided.

**Fig. 4:**
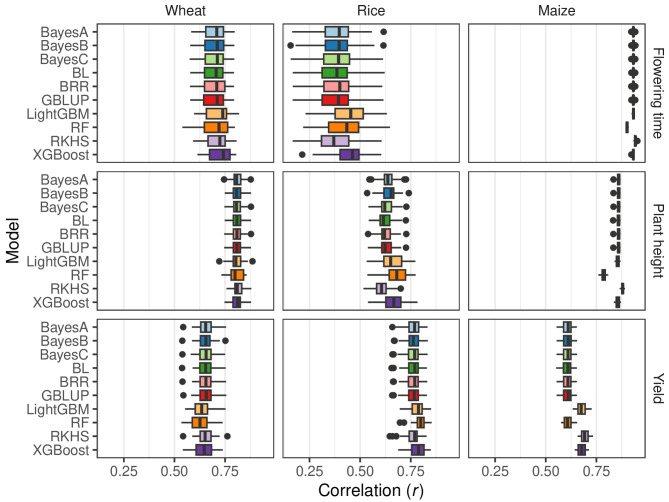
Boxplots showing the Pearson’s correlation coefficient (*r*) between observed and predicted values for 5 × 5-fold cross-validation splits of three traits (plant height, flowering time and yield) in three species (wheat, rice and maize). The *y* axis lists the ten models tested: Bayes A, Bayes B, Bayes C, Bayes Lasso (BL), Bayes Ridge Regression (BRR), Genomic Best Linear Unbiased Prediction (GBLUP), Light Gradient Boosting Machine (LightGBM), Random Forest (RF), Reproducing Kernel Hilbert Spaces (RKHS) and Extreme Gradient Boosting (XGBoost). The *x* axis shows the correlation coefficients. Left panels: Wheat traits with heading date (days after January 1), plant height (cm) and yield (quintals per hectare). Middle panels: Rice traits with flowering time at Faidpur (days), plant height (cm) and panicle number per plant. Right panels: Maize traits with pollen days after planting (days), plant height (cm) and yield (bushels per acre)

The model predictive abilities measured using *r* ranged between − 0.08 and 0.96, with a mean of 0.62. The most predictable trait was ‘density’ for pine, and the least predictable was ‘Awn_presence’ for rice. A multiple linear regression model showed a significant (*p* < 2.2e^−16^, Table S5) overall effect of the predictor variables (crop, trait and model) on the correlation (*r*) between observed and expected in testing data from 5 × 5-fold cross-validation (*r*^*2*^ = 0.87, *p* < 2.2e^−16^). Most of the significant differences (*p* < 2.2e^−16^) originate from species and trait (81/92), as shown in Table S5. A subset of these data are shown in Fig. 4. This was expected as the combinations of trait and species reflects the overall size of the data (number of genotypes and number of markers), which are key factors for determining predictability. Differences between traits are to be expected as they are dependent upon genetics, heritability, developmental stability, environmental factors and genotype-by-environment interaction.

The effects of different models were less clear. For the Bayesian models, no significant difference was found between BayesB, BayesC, Bayesian Lasso, Ridge Regression and GBLUP (*p* > 0.05) compared to BayesA. However hyper parameter tuning was not performed for these models. The model RKHS performed significantly (*p* = 0.004617) worse than BayesA, but the effect estimates were small (*β* = −0.006). For the random forest and two gradient boosting methods, the accuracy was significantly higher (*p* < 1e^−10^). However, the improvement was less than 2.5% for LightGBM (*β* = 0.021), random forest (*β* = 0.014) and XGBoost (*β* = 0.024). But, in most cases, the semi-parametric and non-parametric models compared equally to their Bayesian counterparts, as shown in Fig. 4 for height and yield in maize.

### Resource usage

**Fig. 5:**
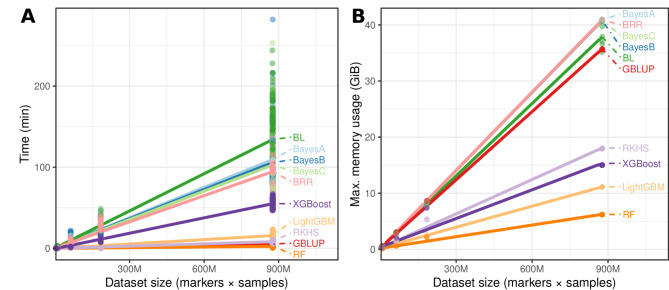
Comparison of model fit (**A**) running time (minutes) and (**B**) maximum memory usage (in gibibytes) for model training for each 5 × 5-fold cross-validation split for various datasets. The models Bayes A, Bayes B, Bayes C, Bayes Lasso (BL), Bayes Ridge Regression (BRR), Genomic Best Linear Unbiased Prediction (GBLUP), Light Gradient Boosting Machine (LightGBM), Random Forest (RF), Reproducing Kernel Hilbert Spaces (RKHS) and Extreme Gradient Boosting (XGBoost) are represented by different colors. Dataset size is indicated by the total number of data points (markers × samples). Linear regression lines show the relationship between each variable and the number of data points. All processing was performed using one CPU thread per dataset and model

Given the models tested were broadly equivalent to each other, we also evaluated resource usage in terms of computational time and maximum memory usage (Fig. 5). Comparison of the user time to fit each model revealed large differences. For the machine learning models, these measurements do not account for the time required for hyperparameter optimization, which is not needed for Bayesian or GBLUP models. The fastest model was random forest in rice for the trait ‘Leaf_pubescence’, which took 0.19 s user time, on average, per split of the 5 × 5-fold cross-validation. In contrast, the BayesB model in maize for the trait ‘Ear_Height_cm’, took around 164 min user time, on average, for each split of the 5 × 5-fold cross-validation. The number of data points (markers × genotypes) best explained (*p* < 2.2e^−16^, *r*^*2*^ = 0.56) the computational user time compared to the number of genotypes (*p* < 2.2e^−16^, *r*^*2*^ = 0.52) and number of markers (*p* < 2.2e^−16^, *r*^*2*^ = 0.52) alone. The user time was clearly dependent upon the model, as shown in Fig. 4, where the Bayesian models took typically an order of magnitude longer than their machine learning counterparts and RKHS. An exception is the GBLUP model, which had user computational times like that of the non-parametric and semi-parametric methods. When inspecting the memory usage, a similar trend emerged, where datasets containing around 900 M data points required over 30 GB of RAM, whilst RKHS, XGBoost, LightGBM and random forest required less than 20 Gb of RAM.

Collectively, different models may offer marginal improvements in predictive performance, but no single model consistently outperforms others across all datasets. However, model selection may be influenced by contextual factors, particularly time constraints. Although computational time is often mitigated by parallel processing, some scenarios, like speed breeding systems [38], breeders can achieve multiple generations per year (up to six in cereals). Such rapid generation cycles require timely decisions between breeding intervals. Consequently, prioritizing faster computational methods for genomic prediction may be justifiable even at the expense of predictive accuracy.

### Usage notes

**Fig. 6:**
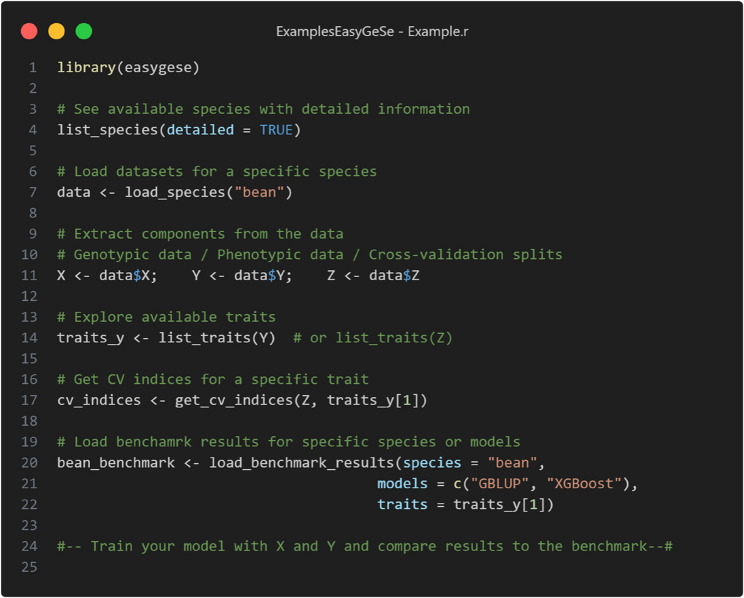
Usage example of the functions in the EasyGeSe R package implementation. In this example the bean dataset is loaded along with the indices and benchmark results for the first trait in the Y object

**Fig. 7:**
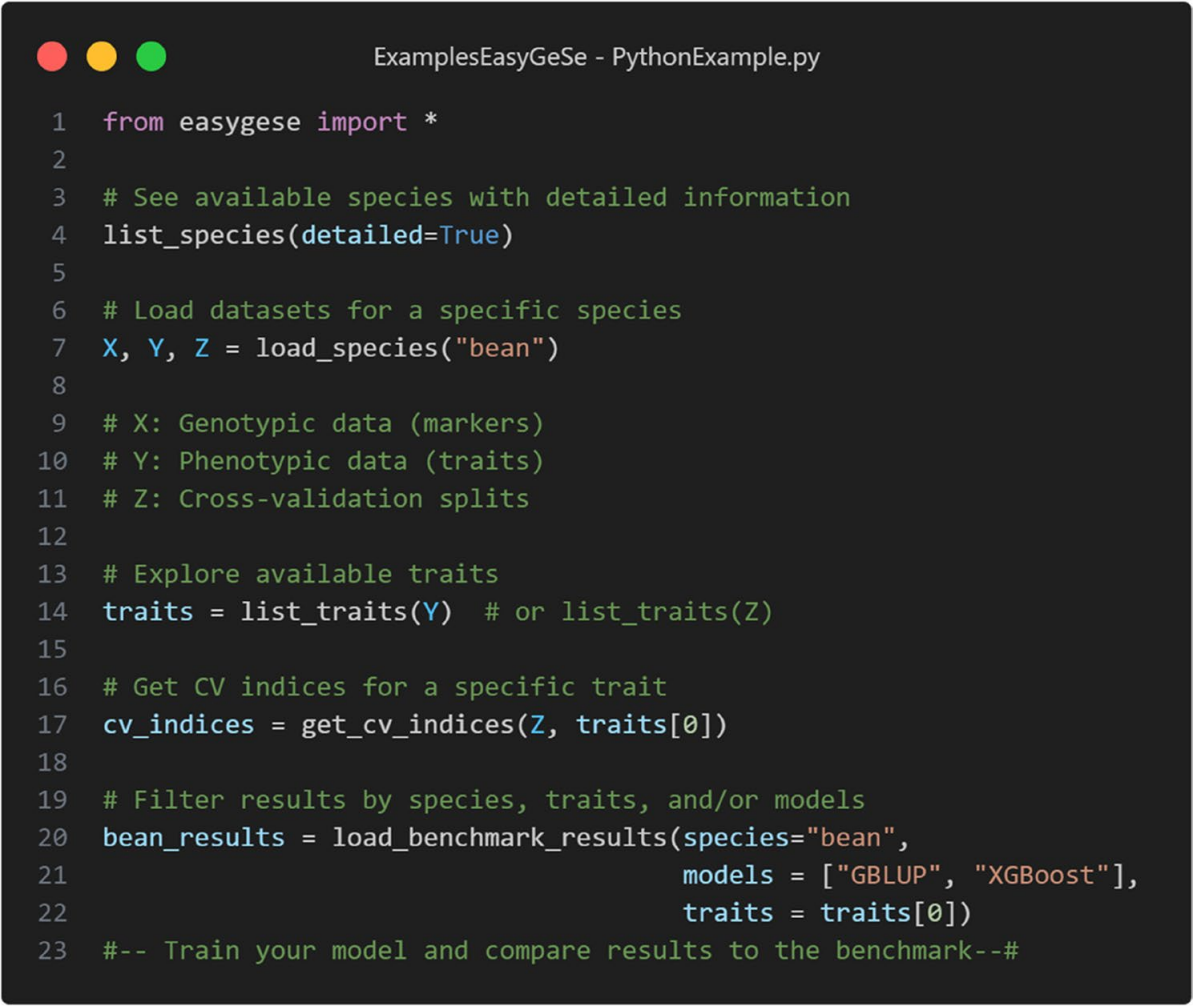
Usage example of the functions in the EasyGeSe Python package implementation. In this example the bean dataset is loaded along with the indices and benchmark results for the first trait in the Y object

The functions to access and navigate the dataset can be installed as a package both in R and Python. An overview of the most important functions and what they do can be found in Table 3. Whilst simplified, in Figs. 6 and 7, we demonstrate how to use the package to load a dataset in R and Python, respectively. Additional information can be browsed in the GitHub page (https://github.com/cquesadat/EasyGeSe/) for this project.

**Table 3:** Important functions implemented in the EasyGeSe packages

Function	Description
list_species()	Lists available species and provides a code snippet to do so
load_species()	Given a species name, loads X, Y and Z objects
get_cv_indices()	Given a Y or Z object, lists all available traits
load_benchmark_results()	Given a Z object and a trait, returns cross-validation indices
download_data()	Given a species name downloads the relevant data locally
load_benchmark_data()	Loads the benchmarked models either averaged or per split

## Conclusions

EasyGeSe is a ready-to-use benchmarking resource to test new genomic prediction methods in terms of accuracy and efficiency in a wide variety of species. The pre-processing and common formatting across datasets facilitates its usability by a broad scientific community.

## Supplementary Information

Supplementary Material 1.

## Data Availability

The datasets generated and analysed for EasyGeSe and the implemented resources for their analysis are available on GitHub (https://github.com/cquesadat/EasyGeSe/). The processed data are available on Zenodo (https://zenodo.org/records/15348871).
